# Feature Selection in Machine Learning for Perovskite Materials Design and Discovery

**DOI:** 10.3390/ma16083134

**Published:** 2023-04-16

**Authors:** Junya Wang, Pengcheng Xu, Xiaobo Ji, Minjie Li, Wencong Lu

**Affiliations:** 1 Department of Mathematics, College of Sciences, Shanghai University, Shanghai 200444, China; 2 Materials Genome Institute, Shanghai University, Shanghai 200444, China; 3 Department of Chemistry, College of Sciences, Shanghai University, Shanghai 200444, China; 4 Zhejiang Laboratory, Hangzhou 311100, China; 5 Key Laboratory of Silicate Cultural Relics Conservation (Shanghai University), Ministry of Education, Shanghai 200444, China

**Keywords:** perovskites, materials design, machine learning, feature selection

## Abstract

Perovskite materials have been one of the most important research objects in materials science due to their excellent photoelectric properties as well as correspondingly complex structures. Machine learning (ML) methods have been playing an important role in the design and discovery of perovskite materials, while feature selection as a dimensionality reduction method has occupied a crucial position in the ML workflow. In this review, we introduced the recent advances in the applications of feature selection in perovskite materials. First, the development tendency of publications about ML in perovskite materials was analyzed, and the ML workflow for materials was summarized. Then the commonly used feature selection methods were briefly introduced, and the applications of feature selection in inorganic perovskites, hybrid organic-inorganic perovskites (HOIPs), and double perovskites (DPs) were reviewed. Finally, we put forward some directions for the future development of feature selection in machine learning for perovskite material design.

## 1. Introduction

**Figure 1:**
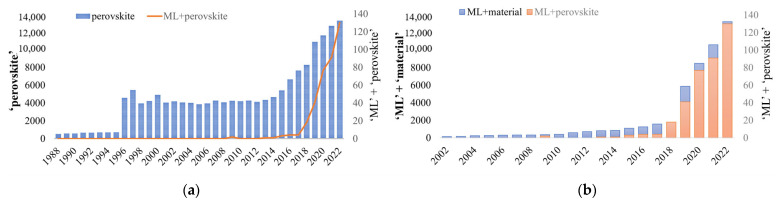
Number of published papers. (**a**) On the key words ‘perovskite’ and ‘machine learning and perovskite’ (from 1988 to 2022). (**b**) On the key words ‘machine learning and material’ and ‘machine learning and perovskite’ (from 2002 to 2022).

Machine learning (ML), as an interdisciplinary technique covering multiple fields of statistics, computer science, and mathematics, has been widely used in the medical, bioinformatics, financial, and agriculture fields [1,2,3,4,5]. Especially in the materials field, ML technology has accelerated the design and discovery of new materials by constructing models for the prediction of their properties [6,7]. In recent years, perovskite materials have drawn the attention of many scholars due to their excellent properties, such as excellent electrical conductivity, ferroelectricity, superconductivity, longer carrier diffusion lengths, a tunable bandgap (*E*_g_), and high light absorption that can be applied in solar cells, light-emitting diodes, lasers, and photocatalysis materials fields [8,9,10,11]. Figure 1a demonstrates the overall growth pattern in the number of papers searched on the website ‘web of science’ with the key words ‘perovskite’ and ‘machine learning and perovskite’ from 1988 to 2022. Especially since 2013, after the breakthrough in the applications of perovskite materials in solar cells, there has been a spurt of research results, indicating that perovskite materials have always been a research hotspot. Figure 1b shows that ML technology has become a powerful tool in materials science in recent years, and its applications in the field of perovskite materials have been increasing year by year since 2013, indicating that ML has played an increasingly important role in the research of perovskite materials.

Data is the cornerstone of ML, and high-quality data allows ML to capture the hidden patterns in the data to make the correct predictions about the research objects. The data of materials are generally divided into target variables reflecting material properties and features associated with the target variables, which can also be described as variables, descriptors, or fingerprints in the material field. For perovskite materials, property data such as *E*_g_, formability, thermodynamic stability, specific surface area (SSA), and Curie temperature (*T*_c_) are commonly employed as target variables, and the associated features usually involve elemental components, atomic parameters, structural parameters, experimental parameters, etc. [12,13,14,15], which usually have the characteristics of high feature dimensionality. The high feature dimensionality would not only lead to limitations due to overfitting and computational inefficiency but also cause difficulty in exploring the physical meaning of features [16,17]. Thus, it is crucial to pick an appropriate method for reducing feature dimensionality. The two commonly used methods for dimensionality reduction are feature extraction and feature selection [18]. Feature extraction transforms the feature space by transformation or mapping, thus effectively reducing the dimensionality of features [19]. Feature selection preserves the original information of features by selecting a valid subset from the original feature set and removing redundant and irrelevant features. Feature extraction may generally lack interpretability, while feature selection methods are numerous. Therefore, it is necessary to select an appropriate feature selection method to approximate the upper limit of the performance of the trained model as much as possible. Reviewing the progress of feature selection methods in ML for perovskite materials and providing an outlook on future work will help further the development of perovskite material design.

In this review, we discuss the applications and importance of feature selection in the ML workflow for perovskite materials. In Section 2, the basic workflow of ML in the field of materials science is outlined. In Section 3, we present the different types of perovskite materials and their associated features. Section 4 is an introduction to feature selection methods, including filter, wrapper, and embedded. In Section 5, the applications of feature selection methods in the study of inorganic perovskites, hybrid organic-inorganic perovskites (HOIPs), and double perovskites (DPs) are introduced. In Section 6, some of the current challenges and opportunities encountered in the applications of feature selection in ML to perovskite design and discovery are briefly discussed. Our work will help researchers better deal with the feature selection problems involved when using ML as a tool to study perovskite materials.

## 2. Workflow of Materials Machine Learning

**Figure 2:**
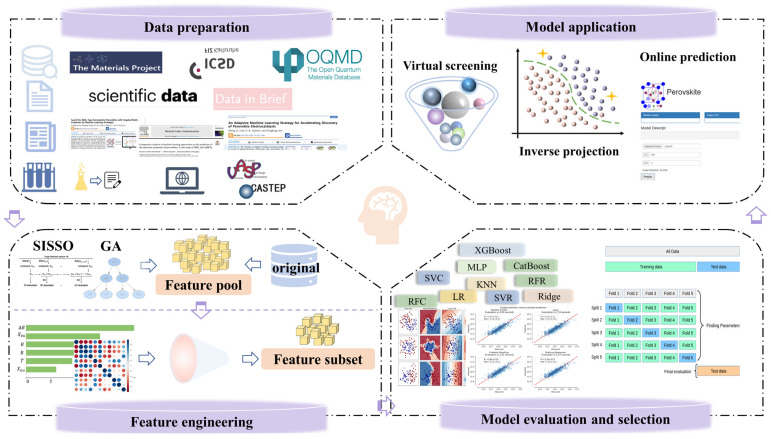
The general workflow of materials ML.

As shown in Figure 2, the workflow of ML in materials could be divided into four steps: data preparation, feature engineering, model evaluation and selection, and model application [20].

Data preparation includes data collection and data preprocessing. Materials data could generally be obtained through publicly available materials databases, published papers, experimental data of the same standard, data journals, and density functional theory (DFT) calculations [13,21,22,23,24]. The latest data can be obtained by searching the literature, but it is time-consuming and laborious. Data from data journals and databases can be obtained in a short time, but the latest data are generally not available in a timely manner. *Scientific Data* by Springer Nature and *Data in Brief* by Elsevier are the more representative data journals. Table 1 lists the commonly used material databases, including perovskites. Experimental data may be a good source of data, but it is costly. DFT calculations are susceptible to material systems, which may lead to the doubling of time and computing resources. Data preprocessing is essential due to the characteristics of multi-source data and the high noise of the material data. To ensure the availability of data, common preprocessing operations include filling in missing values, removing duplicates and outliers, dimensionless processing, treating data imbalances, and rationally dividing data [25,26]. 

Feature engineering, including feature construction and feature selection, is an extremely important part of the ML workflow. In most ML processes, the quality of the data related to the sample size and feature dimensionality, as well as the validity of the features, determines the upper limit of the model’s performance. In general, a high ratio of sample size to feature dimension would lead to better model performance. When the existing features do not contain enough valid information to cause low model performance, new features can be either constructed based on domain knowledge or generated by simple mathematical transformation of existing features through algorithms such as the Sure Independence Screening Sparsifying Operator (SISSO) and genetic algorithm (GA) to improve model performance [27,28]. The properties of materials are influenced by their composition, structure, experimental conditions, and environmental factors, but there may be weakly correlated, uncorrelated, or redundant features in the data. For the original set of features in the data, feature selection can remove the redundant features and keep the key features that are easily accessible and have a significant impact on the target variable to further improve the model’s performance while increasing the computational efficiency.

Before building models, it is necessary to confirm the type of models corresponding to classification and regression models when the target variables are discrete and continuous, respectively. There are many ML algorithms, but no perfect algorithm exists. Although for a specific classification or regression task, the researchers could choose linear, nonlinear, or ensemble algorithms preliminary based on their understanding or guessing of the potential “structure-property relationship” of the materials. It is still difficult to determine the most suitable algorithm based on the limited data volume. Even with the same data and algorithm, the trained model will not be the same with the different hyperparameters. Therefore, it is necessary to evaluate a series of models to select the relatively optimal one. Model performance and model complexity are the key factors that determine model selection. Model performance can be measured by evaluation metrics calculated based on the true and predicted values of the target variable. Common evaluation metrics for regression tasks include coefficient of determination (R^2^), correlation coefficient (R), mean square error (MSE), root mean squared error (RMSE), mean absolute error (MAE), and average relative error (MRE), while common evaluation metrics for classification tasks include accuracy (ACC), area under the curve (AUC), recall, precision, and F_1_ score. To ensure the reliability of the results, the hold-out method and cross-validation method are generally used to evaluate the models after the evaluation metrics are determined. Common methods of cross validation are 5-fold cross validation (5-fold CV), 10-fold cross validation (10-fold CV), and leave-one-out cross validation (LOOCV). Furthermore, we tend to choose the model with better performance and lower model complexity. After selecting a specific ML algorithm, hyperparameter optimization is usually performed to further improve the performance of the model, and the final model is determined after the determination of hyperparameters. Contemporary hyperparametric optimization algorithms can be mainly classified into various types, including grid-search, Bayesian-based optimization algorithms, gradient-based optimization, and population-based optimization.

The final aim of ML is to predict the target variables of unknown samples based on the trained model. The three major scenarios of model application are high-throughput screening (HTS), inverse design, and the development of online prediction programs. HTS uses the constructed model to predict the target variables of a huge number of virtual samples in order to filter out samples with high performance potential and guide experimental synthesis [29,30]. The inverse design can be used to obtain the features of designed samples via the inverse projection method, which is an effective way to realize the material from properties to composition [31,32]. The prediction of designed samples helps screen out candidates with breakthrough performance and improves computational efficiency. The development of an online prediction program makes it possible to quickly achieve the prediction of target properties by simply inputting the necessary information, such as a chemical formula, on the input page, which facilitates the extension of model application and effectively realizes model sharing [33].

## 3. The Structure and Features of Perovskite

Named after Russian geologist Perovski, perovskite can be divided into narrow sense perovskite, referring to the specific compound CaTiO_3_, and broad sense perovskite, referring to the ABX_3_-type compound with the same structure as the compound CaTiO_3_ [34]. The cations at A-site and B-site can be replaced by ions with approximate radii or certain groups due to the tunable ionic structure of perovskite materials, leading to the emergence of a lot of perovskite derivatives. The common perovskites generally can be subdivided into inorganic perovskites, HOIPs, and DPs [8]. The ABX_3_ inorganic perovskites have been widely used in solar cells, solid oxide fuel cells, magnetic refrigeration, and photocatalysis for their multiple properties such as catalytic activity, strong flexibility, outstanding stability, and low cost [8,35]. The HOIPs have been widely applied in the fields of solar cells, light-emitting diodes, X-ray or γ-ray detectors, lasers, and photodetectors due to their longer charge diffusion lengths, high absorption coefficients, high defect tolerance, high carrier mobility, and tunable *E*_g_ [36,37,38]. Because of the adjustable photoelectric performance and good stability, the DPs have demonstrated promising applications in photocatalysis as well as in functional devices including solar cells, light-emitting diodes, scintillators, and photodetectors [39,40].

### 3.1. Inorganic Perovskites

**Figure 3:**
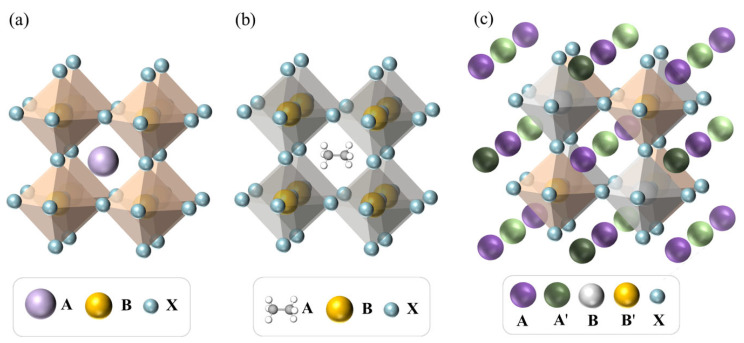
Crystal structures of different perovskites. (**a**) ABX_3_, an inorganic perovskite structure. (**b**) ABX_3_, a hybrid organic-inorganic perovskite structure. (**c**) AA′BB′X_6_, double perovskite structure.

For ABX_3_-type inorganic perovskites, A-site and B-site are cations of alkaline earth or rare earth metal with a larger radius and transition metal with a smaller radius, respectively, and X is usually an anion of oxygen or halogen [41]. As shown in Figure 3a, the ideal structure of ABX_3_ perovskites has cubic symmetry with space group Pm3m, and the cations at the A-site and B-site are coordinated to the X-site anion via 12 and 6, respectively [42]. ABX_3_ inorganic perovskites can be further divided into oxide perovskites and halide perovskites when X refers to oxygen ions and halide ions, respectively. The ABO_3_ perovskite oxides are one of the most common and widely studied structures in materials. Given that not all compounds with ABX_3_ stoichiometry are necessarily perovskite materials, geometric structural features such as the octahedral factor (μ), Goldschmidt’s tolerance factor (t), and a modified tolerance factor (τ) (Equations (1)–(3)) are used in the study of perovskite materials by ML for the determination of perovskite formability and stability [43,44,45]. In addition, the structural features of A-X and B-X bond lengths based on bond valence have also been used to indicate the formability and stability of inorganic perovskites [46]. For ABX_3_-type inorganic perovskites, the features are generally dominated by atomic parameters indicating the properties of the elements in the A/B sites, such as atomic radius, electronegativity, ionization energy, highest occupied molecular orbital (HOMO) energy, and lowest unoccupied molecular orbital (LUMO) energy, etc. It is worth noting that when the elements at the A-site or B-site of the ABX_3_ perovskites are doped, the general formula can be expressed as A_1−x_A′_x_B_1−y_B′_y_X_3_ in which the features of the A/B positions are generally calculated by taking a weighted average of the properties of the doped elements at the corresponding positions (Equations (4) and (5)) [47,48]. Commonly used atomic parameters are publicly available from the Villars database [49], Mendeleev package [50], and RDKit [51] and can also be obtained by direct populating through online calculation platforms or software [33]. The models based on 21 features including structural and atomic parameters of the materials populated by the OCPMDM platform developed in our laboratory have yielded good results in the prediction of target variables such as SSA and *E*_g_ of ABO_3_-type perovskite materials [13]. In addition, the SISSO method can be used to generate new key features based on features that are directly accessible. Equation (3) is a new tolerance factor obtained by Bartel et al. based on the SISSO method of ionic radii, which has an ACC of 92% in determining the formability and stability of ABX_3_ perovskites [45].

(1) $$\mu =\frac{r_{B}}{r_{X}}.$$

(2) $$t=\frac{r_{A}+r_{X}}{\sqrt{2}\left(r_{B}+r_{X}\right)}.$$

(3) $$\tau =\frac{r_{X}}{r_{B}}-n_{A}\left(n_{A}-\frac{\frac{r_{A}}{r_{B}}}{ln\left(\frac{r_{A}}{r_{B}}\right)}\right).$$

(4) $$f_{\bar{A}}=\left(1-x\right)*f_{A}+x*f_{A^{\prime }}.$$

(5) $$f_{\bar{B}}=\left(1-y\right)*f_{B}+y*f_{B^{\prime }}.$$

where $r_{A}$, $r_{B}$, and $r_{X}$ are the ionic radii of ABX_3_ perovskites, respectively; $n_{A}$ is the oxidation state of the A-site ion; (1−x) and x are the percentages of A-site doped elements, (1−y) and y are the percentages of B-site doped elements; $f_{A}$ and $f_{A^{\prime }}$ are the respective features of A-site doped elements, and $f_{\bar{A}}$ is the weighted average feature of the A-site; $f_{B}$ and $f_{B\prime }$ are the respective features of the B-site doped elements, and $f_{\bar{B}}$ is the weighted average feature of the B-site.

### 3.2. Hybrid Organic-Inorganic Perovskites

As shown in Figure 3b, the A-site of ABX_3_-type HOIPs could be replaced by an organic cation such as methylammonium CH_3_NH_3_^+^ or formamidinium CH(NH_2_)_2_^+^ compared to ABX_3_ inorganic perovskites [52]. The features involved in the inorganic part of the HOIPs are still dominated by atomic parameters, but the organic molecular features have few parts in common with the atomic features due to the complexity of the organic cation at the A-site, which requires additional calculations of the features of the organic structure [53,54]. The basic properties of an A-site ion, such as its first and second ionization energies, electron affinity, molecular volume, molecular radius, and chemical potential, can be estimated based on theoretical methods, and Multiwfn and Gaussian are commonly used calculation software [31,53]. In addition to using the radius of organic ions as a feature, the anisotropy of organic cations can also be considered. Chen et al. improved the ACC of *E*_g_ models by using three geometric parameters, namely length, width, and height, as features [55].

### 3.3. Double Perovskites

The structural general formula of DPs could be expressed as AA′BB′X_6_, where A and A′ are more commonly the same or different cations, and B and B′ are different cations that alternate with the X site ions to form the BX_6_ and B′X_6_ octahedrons (Figure 3c) [56,57,58]. Similarly, not all materials satisfying the chemical formula AA′BB′X_6_ are perovskite structures. The tolerance factor t is proposed for single perovskite materials, but the formability of perovskites is essentially all based on geometric criteria derived from ion radii or bond distances. By using arithmetic or weighted averages of ion radii or bond lengths, the concept of tolerance factors can be extended to DPs with more complex compositions [12]. The generalized octahedral factor has also been introduced as a judgment of perovskite formability [39]. For DPs, the common features are similar to those of the ABX_3_ type, which are generally based on atomic parameters. And similar to single perovskites containing doped elements, the features of the A/B sites can be treated by common methods including arithmetic averaging and geometric averaging [59]. It is also noteworthy that AA′BB′X_6_, A′ABB′X_6_, and A′AB′BX_6_ are all unified systems because the exchange of two A-site cations as well as two B-site cations does not affect the structure of perovskite. The features are treated symmetrically when considering the inclusion of structural symmetry into the model [56].

Furthermore, experimental conditions are also quite important features, and the gradient boosting regression (GBR) model for the crystallite size (CS) of ABO_3_ perovskite materials developed by Tao et al. indicates the high importance of two experimental conditions: the preparation method (PM) and the calcination temperature (CT) [15]. If possible, it is encouraged to use the experimental conditions as features to build predictive models for the target properties.

Notably, perovskite materials are widely used in solar cells and photodetectors in the form of thin films [36]. Especially in the field of solar cells, the power conversion efficiency (PCE) of perovskite solar cells (PSCs) has surpassed 25% within just 10 years, which is comparable to crystalline silicon solar cells [60]. Research has revealed that high-quality thin films are one of the crucial factors influencing the performance of PSCs. The methods to fabricate perovskite films include several techniques such as solution processing, vacuum deposition, physical vapor deposition, vapor-assisted solution processing, and scalable deposition [61,62,63,64,65,66]. Thin film properties such as grain size, morphology, crystallinity, defect density, and surface coverage may vary under different preparation methods, leading to differences in the quality of the thin film [61]. Experiments have shown that various process parameters such as stoichiometry, thermal treatment, substrate temperature, solvent engineering, additives, and environmental control have a great influence on the quality of perovskite thin films [61,62,63,64,65,66].

## 4. The Methods of Feature Selection

According to whether the evaluation criteria are independent of the learning algorithm, the feature selection methods could be generally classified into filter, wrapper, and embedded [67,68]. The filter methods are independent of the ML algorithm, using an evaluation criterion based on statistical theory or information theory to select a subset of features after ranking the features [19,69]. In the process of feature selection, the wrapper methods use the performance of the evaluator as the criterion to select the optimal feature subset [70]. The embedded methods can be used to realize feature selection in the modeling by combining the training of the evaluator and the processes of feature selection into a single optimization process [17]. The filter methods are computationally efficient and generalize well. However, due to the lack of interaction with the evaluator, the model performance of feature subsets selected based on the filter methods is generally less effective than the wrapper and embedded methods, which are relatively less computationally efficient [71].

### 4.1. Filter

The filter feature selection methods include the chi-square test ($\chi^{2}$), analysis of variance (ANOVA), Pearson correlation coefficient (*PCC*), distance correlation coefficient (DCC), max-relevance and min-redundancy (mRMR), maximal information coefficient (MIC), and Relief, etc. 

The $\chi^{2}$ and ANOVA are correlation measure methods based on hypothesis testing, with the former for testing the independence between discrete variables and the latter for testing the independence between discrete and continuous variables [72,73]. Hypothesis testing generally includes four steps: (1) proposing the null hypothesis and alternative hypothesis; (2) designing the hypothesis testing statistic according to the hypothesis; (3) getting the *p*-value according to the distribution after calculating the current value of the statistic; and (4) considering the acceptance or overturning of the null hypothesis according to the *p*-value and drawing the final conclusion. The smaller the *p*-value of the output, the smaller the probability that the null hypothesis holds, and the more likely it is that the two features are not independent. Features with significant associations can be screened out when the *p*-value is less than α referring to the significance level. It is worth noting that, generally, the smaller the *p*-value usually means the larger the value of the statistic, which can be equated to the feature score. In specific usage scenarios, the user can select features based on the ranking of features according to the value of the statistic [74].

*PCC* generally measures the linear correlation between continuous variable pairs (x,y) by Equation (6), where n is the number of samples in the dataset, $x_{i}$ and $y_{i}$ are the *i*th sample point, and $\bar{x}$ and $\bar{y}$ are the means of the samples [75,76]. The range of *PCC* values from −1 to 1 indicates that the relationship between variables changes from a completely negative correlation to a completely positive correlation. Additionally, the closer the *PCC* is to zero, the weaker the linear correlation will be. In a practical ML process, *PCC* can indicate the linear correlation between the target variable and features to represent the degree of association and a linear correlation between any feature pairs to represent the redundancy among feature pairs:

(6) $$PCC=\frac{\sum_{i=1}^{n}\left(x_{i}-\bar{x}\right)\left(y_{i}-\bar{y}\right)}{\sqrt{\sum_{i=1}^{n}{\left(x_{i}-\bar{x}\right)}^{2}}\sqrt{\sum_{i=1}^{n}{\left(y_{i}-\bar{y}\right)}^{2}}}.$$

The accuracy of *PCC* may not be guaranteed when there is a nonlinear correlation between the variables. The DCC is an alternate correlation coefficient that does not have this weakness, which defines the independence between variables: dCor(x,y)=0 if and only if x and *y* are independent, where dCov(x,y) is the sample distance covariance (Equation (7)) [77]. The DCC takes a value in the range [0, 1]; the larger the value, the stronger the correlation:

(7) $$dCor\left(x,y\right)=\{\begin{array}{c} \frac{dCov\left(x,y\right)}{\sqrt{dCov\left(x\right)dCov\left(y\right)}},\;dCov\left(x\right)dCov\left(y\right)>0\\ 0,\;\;\;\;\;\;\;\;\;\;\;\;\;\;\;\;\;\;\;\;\;\;\;\;\;dCov\left(x\right)dCov\left(y\right)=0 \end{array}.$$

The measure of correlation based on mutual information is a non-parametric approach, and the essence of mutual information is the extent to which two variables explain each other, which can be understood in terms of the consistency of the distribution and the amount of information contained in each other. Meanwhile, mutual information can identify arbitrary relationships between any type of variable.

mRMR based on mutual information theory attempts to select the features with the maximum relevance to the target variable and the minimum redundancy among the features [78]. It is supposed that there are a total of F features in the dataset, and $S_{m}$ denotes the set of m features that have been selected; the importance of the (*m* + 1)th feature is defined in Equation (8), where $I\left(x_{j},y\right)$ is the mutual information between variables $x_{j}$ and y. Additionally, the mutual information of any variable pair (x,y) could be calculated by Equation (9), where p(x), p(y), and p(x,y) are their probabilistic density functions. Then the scoring function $\begin{array}{c} max\\ x_{j}\epsilon \left(F-S_{m}\right) \end{array}\left[f^{mRMR}\left(x_{j}\right)\right]$ can be used to select the (*m* + 1)th feature from the remaining set of features $\left(F-S_{m}\right)$ to join $S_{m}$. Therefore, mRMR is actually a stepwise method where, at each step of the feature selection process, the feature with the highest feature importance will be added to the subset until the number of features in the subset reaches the user requirement:

(8) $$f^{mRMR}\left(x_{j}\right)=I\left(x_{j},y\right)-\frac{1}{m}\sum_{x_{i}\epsilon S_{m}}I\left(x_{j},x_{i}\right),$$

(9) $$I\left(x,y\right)=\iint p\left(x,y\right)\log\frac{p\left(x,y\right)}{p\left(x\right)p\left(y\right)}dxdy.$$

The solution of joint probabilities is often difficult, and MIC overcomes this shortcoming of mutual information. The MIC belonging to the nonparametric method can provide an effective measure of linear and nonlinear relationships between the variables, as well as nonfunctional dependencies [79]. The values of MIC between the features and the target variable are regarded as the scores of each feature in the feature selection process. The features can be ranked based on the sizes of the MIC values, and the features are then chosen based on the threshold value or the predetermined number of features.

Relief is a feature weighting method used to handle binary classification, where features are given different weights according to the relevance of each feature to the category, which is based on the ability of the feature to discriminate between nearby samples, and features with weights less than a certain threshold are removed [69]. According to regression and classification tasks, the ReliefF and RReliefF methods were proposed, which support multi-class classification and regression problems, respectively [69].

### 4.2. Wrapper

Wrapper methods for feature selection include greedy sequential searches such as sequential feature selection (SFS) and sequential backward selection (SBS), as well as more complex ones like recursive feature elimination (RFE) and evolutionary and swarm intelligence algorithms such as GA [80,81,82].

The SFS method takes the empty set as the starting point of the search and selects one feature at a time that makes the objective function generally optimal, referring to the cross-validation score of an estimator to join the feature set S. The SFS selection method is an iterative selection process that involves only adding features. In contrast to SFS, the SBS method starts with the full set of features and then continuously discards features from the feature set to optimize the objective function value. Both methods stop searching when a set number of features is reached.

RFE is a feature selection method based on model performance that continuously removes the least important features through recursion. The basic execution steps of RFE are: (1) training on the current feature set $S_{1}$ and calculating the importance of each feature according to the given evaluator; (2) eliminating the least important feature to obtain the feature subset $S_{2}$, and then training the model again to calculate the importance of the remaining features; and (3) repeating step two until the number of features is equal to the value manually set. The recursive feature addition (RFA), the opposite method, iteratively adds features [83]. RFE and RFA are often used in conjunction with the RF algorithm [83,84].

GA as one of the representatives of intelligent algorithms is proposed based on the core idea of biological evolutionary theory, where each solution is encoded as a ‘chromosome’ or an individual to constitute a population (a subset of all possible solutions) when solving a problem [85]. The general steps of GA include: (1) generating an initial population representing potential solutions to the problem randomly; (2) selecting an appropriate fitness function to evaluate individuals; (3) then applying genetic operations such as selection, crossover, and mutation to generate new populations; and (4) repeating steps 2–3 until the termination condition of the iterative calculation is met [86,87]. Binary coding is adopted when using GA to solve the problem of feature selection, where a binary value of ‘1’ indicates that a feature at the corresponding position is selected, so that a genetic individual consisting of a fixed-length binary string represents a subset of features [87]. In other words, the realization of feature selection based on GA is to find an optimal binary code which represents the optimal feature subset.

### 4.3. Embedded

Embedded methods can be broadly classified into those based on regularized models and those based on tree models. Many ML models introduce regularization terms such as $L_{1}$-penalty or $L_{2}$-penalty in their loss functions to prevent overfitting problems. Regularization terms such as least absolute shrinkage and selection operator (LASSO), ridge regression (RR), and support vector machine (SVM) can effectively shrink the coefficients of certain features to zero, thus enabling feature selection [88,89,90]. A major branch of ML is tree-based ML models such as random forest (RF), GBR, and extreme gradient boosting (XGBoost), etc. [91,92,93]. These tree models record how each feature progressively reduces the model error in the bifurcation of the tree nodes during the process of modeling and generally use feature importance to indicate the degree of feature contribution to the current model.

In addition, SHapley Additive exPlanations (SHAP) method, which can be used in nesting with different ML algorithms, serves as a unifying framework for interpreting black box models, and the SHAP value also indicates how much the feature contributes to the model’s prediction. Since global importance is required, the average of the absolute Shapley values for each feature is used as the SHAP feature importance. Then feature selection can be performed after ranking the features according to SHAP feature importance [94].

## 5. Feature Selection in Machine Learning for Perovskite Materials

### 5.1. Feature Selection for Inorganic Perovskites

In the research of inorganic perovskite materials, a single feature selection method was sometimes employed. Priyanga G et al. [95] used ML methods to predict the nature of *E*_g_ of ABO_3_ perovskite oxides. Datasets were obtained from various databases and experimental research papers, with the features generated using Matminer. After preprocessing, 5276 samples consisting of ‘direct *E*_g_’ and ‘indirect *E*_g_’ were obtained to construct the classification model for predicting the nature of *E*_g_ in perovskite materials. The highly correlated features were removed based on the *PCC* matrix, retaining the six features, including the ionic radius of the A-site (*R*_A_), the ionic radius of the B-site (*R*_B_), the electronegativity of element A (*E*_NA_), the electronegativity of element B (*E*_NB_), the electronegativity difference with radius (*E*_NR_), and the average ionic character of A and B (avg ionic char [95]. Logistic regression (LR), decision tree (DT), RF, k-nearest neighbors (KNN), light gradient boosting machine (Light GBM), XGBoost, and support vector clustering were used to build the classification models, and the RF model was optimal with an ACC of 91%. A feature importance analysis of the RF model revealed that the most important features in the *E*_g_ classification of perovskite materials are avg ionic char *E*_NA_, *E*_NB_, and *E*_NR_. Additionally, the tendency to obtain direct *E*_g_ is higher as the average ionic character increases, while the tendency to obtain indirect *E*_g_ increases as the average ionic character decreases. Zhang et al. [96] developed a model for the automatic identification of perovskite crystal structures. Firstly, 1647 ABX_3_-type perovskites data containing seven crystal systems, 40 space groups, and lattice parameters were extracted from the MP database, and the initial features include 24 elemental and structural descriptors. The recursive feature descriptor method was used in the feature engineering process to eliminate weakly and unreliably correlated atomic parameters while maintaining the same level of model accuracy. Ten features, including the number of atoms, bond-valence vector sum (BVVS), and atomic number (Z), were ultimately kept. The SVM, RF, gradient boosting trees (GBDT), and XGBoost algorithms were used in combination with the selected features to build classification and regression models, respectively. The RF model did the best when the models were first built using a subset of features without BVVS. Subsequently, the RF was used to build classification and regression models based on a subset of features containing BVVS. Additionally, the ACC of the crystal systems classification model increased from 0.915 to 0.974, and the R^2^ of the lattice constant model increased from 0.710 to 0.887, indicating that the addition of BVVS can more accurately reflect the structural properties of crystals.

**Figure 4:**
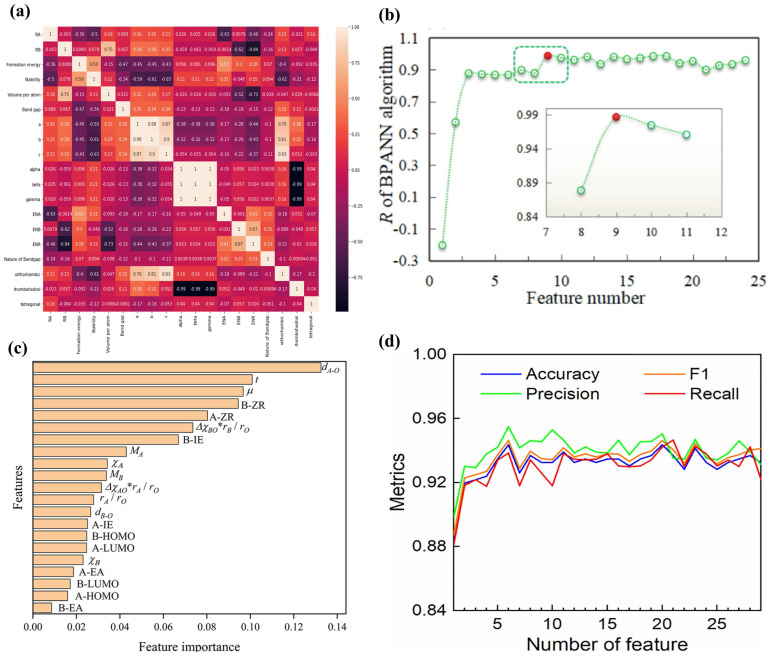
Applications of feature selection in inorganic perovskites. (**a**) A heat map of the correlation between features. Reprinted with permission from ref. [95]. Copyright 2022 Elsevier. (**b**) For R of the LOOCV in the feature selection process of the $R_{H_{2}}$ model, the position of the red point is the maximum value of R. Reprinted with permission from ref. [30]. Copyright 2021 Elsevier. (**c**) The feature importance of the 21 features in predicting the formability. Reprinted with permission from ref. [46]. Copyright 2022 The Authors. (**d**) An evaluation index vs. feature number of ABX_3_ compounds based on the recursive elimination method. Reprinted with permission from ref. [59]. Copyright 2021 Elsevier.

*PCC* as the ‘star method’ for feature selection is also often used as a feature primary screen or mixed with other feature selection methods such as mRMR, RFE, and embedded methods. In addition, there are other different feature selection methods that are mixed or used step by step. Zhao et al. [46] used the ML method to screen formable and stable perovskite oxides from unexplored ABO_3_ combinations. The input data for the ML model consisted of 343 known ABO_3_-type perovskites and 21 initial features. Feature selection was performed based on feature correlation and importance to remove redundant and less important features. Feature correlation was measured by the *PCC* method, and paired Pearson correlation coefficients (*PCC*s) were calculated for the 21 features. Feature importance was obtained from the results of 100 RF models for formability and stability prediction. The importance of the features demonstrates that the formability of perovskites depends mainly on the structural features of the A- and B-site elements, while the properties of the B-site element are the key factor to predict the stability of perovskites. Finally, 16 features were retained for training the formability and stability prediction models of perovskites by analyzing their correlation and importance. For comparison, the RFE method was also used to evaluate the importance of 21 features, and 17 features were retained. The prediction models for formability based on 21, 16, and 17 features, respectively, were denoted as models 1–3, and model two had the highest ACC, precision, F_1_ score, and AUC with 0.988, 0.983, 0.992, and 0.999, respectively. Additionally, 21, 16, and 17 features were combined with E-hull to train the stability prediction models, which were denoted as models 4–6, and model five had the best overall results with an AUC as high as 0.983. Li et al. [59] also studied the formability of perovskites based on ML. First, 576 ABX_3_-type compounds, including 314 perovskites and 262 non-perovskites, were collected from publications. The initial features were 53 physicochemical parameters. In the step of feature engineering, the initial screening of features was first performed based on the *PCC* method, and the number of feature dimensions was reduced to 29 by using 0.9 as the selecting threshold. For further feature selection, the RFE method was applied to the 29 features, and finally six features (τ, μ, t), the ratio of A ion radius to B ion radius ($R_{A}/R_{B}$), Pauling electronegativity (EP_A), and dipole polarizability of the B-site (DP_B), were retained. Subsequently, five ML algorithms, including RF, DT, SVM, KNN, and LR, were used to construct the classification models, of which the RF model was optimal and the ACC of the model after hyperparameter optimization reached 94.85%. Moreover, it was found that the RF model also correctly predicted whether the compounds could form DPs after testing. The importance of the features of the model shows that τ plays a decisive role in the classification model to distinguish between perovskites and non-perovskites. Tao et al. [30] accelerated the discovery of new high-performance and low-cost perovskite photocatalysts in the field of photocatalytic hydrolysis (PWS) by building ML models for hydrogen production rate ($R_{H_{2}}$) and *E*_g_. First, 160 ABO_3_ perovskite photocatalyst data were collected from the experimental literature, of which the $R_{H_{2}}$ and $E_{g}$ datasets contain 77 and 124 samples, respectively. For the *E*_g_ model, the initial features are 17 atomic parameters and three experimental conditions, while there are 18 atomic parameters and six experimental conditions for the $R_{H_{2}}$ model. Four algorithms, including GBR, support vector regression (SVR), backpropagation artificial neural network (BPANN), and RF, were used to construct the regression models. The mRMR method was used to select the best subset of features for the SVR and BPANN models, while the embedded method was used for the GBR and RF models. The BPANN and GBR models performed optimally for $R_{H_{2}}$ and $E_{g}$ prediction, which correspond to feature subset dimensions of 9 (Figure 4b) and 7, respectively, while the R of LOOCV reached 0.9869 and 0.9217. Subsequently, Tao et al. [15] proposed a stepwise design strategy for multi-objective optimizations to accelerate the design of potential ABO_3_ perovskites with high photocatalytic activity. Data were obtained from the published experimental literature, where the sample sizes used to build models for *E*_g_, SSA, and CS were 170, 172, and 117, respectively, and the features included 20 atomic parameters and three experimental conditions. Preliminary feature selection was performed by combining *PCC* and mRMR methods to remove highly correlated features. Firstly, the features of *E*_g_, SSA, and CS were ranked using the mRMR method. Then the *PCC*s of any feature pairs were calculated, and if the value of the *PCC* was greater than 0.9, the features with a lower ranking of mRMR were removed. After the initial selection, *E*_g_ model retained 19 features, while the SSA and CS models both retained 20 features. GBR, SVR, BPANN, and multiple linear regression (MLR) were used to construct the models. The results of LOOCV indicated that GBR was the optimal model with an R of 0.8869 and 0.8733 for predicting *E*_g_ and CS, while SVR was the optimal model with an R of 0.8461 for predicting SSA. In further feature selection, the embedded and mRMR methods were used to select the best features for the GBR and SVR models, respectively, and the final number of retained features was 6, 10, and 9 for *E*_g_, SSA, and CS, respectively. The SHAP analysis of the retained features showed that the boiling point of the B site showed a significant positive correlation with *E*_g_ and contributed the most to the GBR model; the CT and electron affinity of the B site were key features for the SVR model of SSA; and for the CS model, the CT showed a significant positive correlation with CS, which is consistent with the actual experimental conclusion that the higher the CT, the larger the CS formed.

Some researchers had used a particular feature selection method as a tool to determine whether the initial feature subset was valid and then taken other measures to construct other, more useful features. Liu et al. [28] collected 3430 samples to predict the formation of the oxygen vacancy defect in perovskites. The target variable is the oxygen vacancy formation energy, which is defined as a dichotomous problem of whether an oxygen vacancy defect is likely to form or not by using 0.5 eV as the cutoff, and the initial features are 16 structural parameters containing ionic radius, ionic chemical valence, electronegativity, lattice parameters, tolerance factor, and octahedral factor. In the feature engineering, after drawing the correlation coefficient heat map of the features and the target variable, it was found that no feature was significantly correlated with the target variable; therefore, symbolic classification is used to discover the hidden underlying physical relationships. Since the parsimony coefficient can change the complexity of the corresponding formulas of the generated new structural features, a parsimony coefficient of 0.01 was chosen after weighing, and a simple and effective new structural descriptor, $n_{a}\left(r_{a}/E_{na}-r_{b}\right)$, was obtained, with the $n_{a}$, $r_{a}$, and $E_{na}$ meaning the valence, radius, and electronegativity of the a-site ion, respectively, and $r_{b}$ being the b-site ion radius. After modeling with the newly constructed descriptor, the AUC of the interpretable model could reach 0.797. Talapatra et al. [12] constructed their ML model to predict the formability and thermodynamic stability of perovskites. Firstly, a database *D*_F_ of formability and a database $D_{S}$ of thermodynamic stability of perovskite were established. *D*_F_ consists of experimentally known ABO_3_ and AA′BB′O_6_ types of perovskites collected from the literature, including 1187 perovskites and 318 non-perovskites. *D*_*S*_ contains 3469 samples from their own, independently constructed, basic chemically compatible dataset *D*_C_. It was found that 1501 perovskites are thermodynamically stable, while the remaining 1955 are thermodynamically unstable after being calculated by DFT. Structural and chemical features were initially used. These features are associated with the A- and B-site atoms of single perovskites, the A-, A′-, B-, and B′-site atoms, and the symmetric and antisymmetric compound features of DPs. The RFE method was used for feature selection. It was found that atomic features, electron affinity, and geometric features had significant effects on formability and stability. 24 constructed symmetric and antisymmetric compound features based on the first six features and 4 geometric features, including *t*, μ, and mismatch factors ($\bar{\mu_{B}}$ and $\bar{\mu_{A}}$) were finally retained. For the formation and thermal stability of perovskites, RF classification models were constructed based on these 28 features, respectively, and the average classification ACC reached 94.01% and 94.09%, respectively. The analysis of feature importance reveals that not only the traditional t and μ contribute very highly to formability, but also many elemental features at the B-site, such as the Zunger pseudopotential radius, electronegativity, and LUMO, are important features to distinguish perovskites from non-perovskites. For the stability classification model, the symmetry features of B-site, such as HOMO, LUMO, ionization energy, and pseudopotential radius, are key features, and the t is the most important among the geometric features. There is an interesting phenomenon that RFE is the most common feature selection method in the ML workflow for predicting formability and stability. In some application scenarios, GA is also a more effective feature selection method. Xu et al. [13] proposed a multi-properties ML strategy to accelerate the discovery and design of ABO_3_-type ferroelectric perovskites. The data were obtained from publications, including classification data containing 86 ferroelectric perovskites and 61 non-ferroelectric perovskites and regression data containing 95 SSA, 185 *E*_g_, 110 *T*_c_, and 29 dielectric loss (tanδ) samples. A total of 21 atomic parameters were selected as initial features, and seven features were retained using GA combined with the support vector classification (SVC) model for feature selection. The prediction ACC of LOOCV of the SVC model after hyperparameter optimization was increased from 85.59% to 87.29%. Regression models for SSA, *E*_g_, *T*_c_, and tanδ were built based on the ML workflow and SISSO method, respectively. The SSA, *E*_g_, *T*_c_, and tanδ models by ML workflow all used GA and SVR to select features, and the number of retained features were 13, 16, 16, and 2, respectively. The LASSO models are constructed by using new features selected by the SISSO method. The analysis results indicated that SSA, *E*_g_, and *T*_c_ tended to be built as regression models by the ML workflow, which had higher R values of 0.935, 0.891, and 0.971, respectively, while a better tanδ model was obtained when using the SISSO method with an R value of 0.931. It could be speculated that the SISSO method may perform better in the case of small datasets. SHAP analysis of the retained features revealed that the three models for SSA, *E*_g_, and *T*_c_ contained nine common features, including six features associated with the A-position, two features associated with the B-position, and molecular mass. The A-site atomic density showed a strong negative association with SSA and *E*_g_, and the B-site atomic density demonstrated a negative correlation with all three target variables, according to the Pearson correlation analysis based on the nine features and target variables.

### 5.2. Feature Selection for Hybrid Organic-Inorganic Perovskites

**Figure 5:**
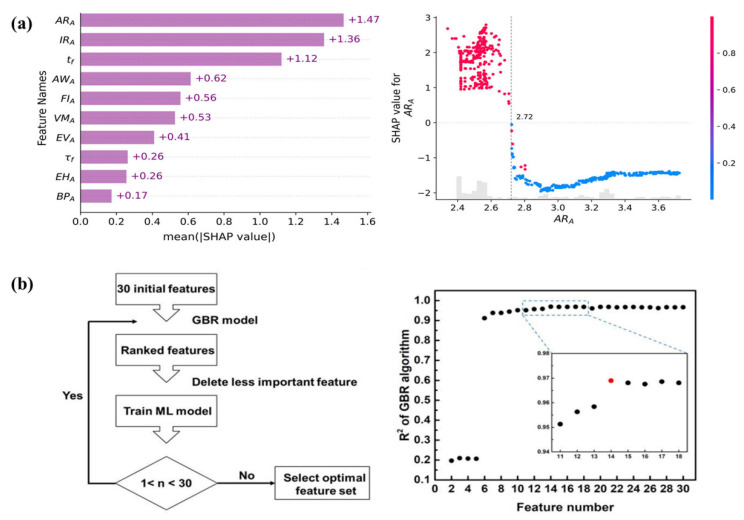
(**a**) Feature importance extracted via the SHAP method, the scatter plot of *AR*_A_, and its SHAP value. Reprinted with permission from ref. [26]. Copyright 2022 American Chemical Society. (**b**) The workflow of ‘last-place elimination’, R^2^ of the GBR model, in each selection process. Reprinted with permission from ref. [97]. Copyright 2018 Springer Nature.

In the study of HOIPs and double HOIPs using ML methods, feature selection by a combination of *PCC* and embedded methods seems to be common. Chen et al. [55] achieved the accelerated discovery of double HOIPs (DHOIPs) by combining ML techniques, HTS, and DFT calculations. The two input datasets consist of 11,161 DHOIPs or HOIPs with *E*_g_ as the target property and 26 initial features, considering the anisotropy of the organic cations at the A-site as well as the HOMO-LUMO gaps and the rotational temperatures. Feature selection was performed based on *PCC* and feature importance from the GBR model, which measured permutation importance and the mean decrease in impurity (MDI). The correlations show that the *R*_A_ and length (*L*_a_) of the A-site cations are highly correlated, and the HOMO-LUMO gap is negatively correlated with the cation size. The GBR model was based on 26 initial features, where both the MDI and permutation importance of the *E*_g_ model with the total dataset as input indicate that the features of B-site play a key role in predicting *E*_g_. Additionally, the accuracy of the model fitted using only the second dataset was very high, with a MAE of only 0.09 eV. Taking *PCC* and feature importance into consideration together, the length *L*_a_ of the A site and the number of f electrons in the B site were finally removed, and 24 features were retained. Lu et al. [26] predicted the experimental formability of HOIPs via imbalanced learning. A total of 539 HOIPs and 24 non-HOIPs were obtained from reported literature as a dataset, while 129 features were created based on the Python package for fast-machine-learning. A total of 43 features were kept after the initial feature selection process, which eliminated constant and strongly correlated features. Nine sampling methods and 10 algorithms were used to handle the imbalanced problem and build the classification models, respectively, and it was found that both combinations of SMOTEENN-CAT and SMOTEENN-SVC achieved 100% ACC and precision of LOOCV after a comparative analysis. The CAT model was nested with the SHAP method to achieve further feature selection, and the highest ACC was achieved for both LOOCV and the test set with 100% and 95.5%, respectively, when the number of features was 28. After analyzing the SHAP feature importance and the relationship between the feature values and the corresponding SHAP values, it is found that perovskite is more likely to be formed when the values of the A site atomic radii (*AR_A_*) are in the range of 2.30–2.72 Å, which can be confirmed by the existing perovskites (Figure 5a). It is also found that both larger *R_A_* and t contribute negatively to the formability of HOIPs.

Moreover, the combination of *PCC* and recursive methods is also very popular among researchers. Zhang et al. [54] predicted the formability of HOIPs using an interpretable ML strategy. A total of 44 HOIPs and 58 non-HOIPs were collected from publications, and raw features consisted of the three structural parameters t, τ, and μ as well as features obtained from the Mendeleev library and Villars database. A two-step method was used to perform the feature engineering, and the first step used the filter method. The number of features was reduced from 339 to 45 after the removal of features with missing values and relatively unimportant features in feature pairs where the *PCC* values exceeded 0.95. Recursive feature addition (RFA) is used in the second stage of the feature selection process to screen out the key features by evaluating the performance of models constructed by the top 2–20 features, which are in the specified feature importance order. For the different algorithms, the specified feature importance is obtained based on the SHAP and mRMR methods, respectively, where the former corresponds to the XGBoost and gradient boosting classifier (GBC) and the latter corresponds to the SVC and the KNN. The optimal prediction ACC under LOOCV was 0.94, 0.91, 0.90, and 0.83 for XGBoost, GBC, SVC, and KNN models with six, four, four, and three features, respectively. SHAP analysis revealed that the *R*_B_ was most important for the formability of HIOP. Wu et al. [98] combined ML techniques and first-principles calculations to achieve rapid screening of mixed double HIOPs (MDHOIPs) for solar cells. Structure-formability classification, *E*_g_ classification, and *E*_g_ regression models were trained based on the reported data of 2274 DHOIPs, with the initial feature set consisting of 87 features related to ion radius, electronegativity, and ion polarizability. Last-place elimination was used to perform feature selection, based on which the relative importance ranking of features can be obtained. For the structure-formability of perovskites, the performance of the classification model was no longer improved when the number of features was greater than 16. The GBC model with an AUC value of 94.3% was trained using the best 16 features, where the ion radius significantly influences the formability of DHOIPs. For the classification and regression models of *E*_g_, the seventh and eighth most important features were selected, respectively. The *E*_g_ classification model had an AUC value of 97.8%, and the GBR model had an R^2^ of 0.974. Both types of models together revealed the importance of the B/B′ site ion, and the GBR model demonstrated that the *E*_g_ value was also influenced by the interaction between the B/B′ site ion and the X site ion. Cai et al. [99] hastened the discovery of novel lead-free hybrid organic-inorganic DPs with excellent stability, a high Debye temperature, and a suitable *E*_g_ for high-performance PSCs based on DFT and ML techniques. The dataset includes 4456 hybrid organic-inorganic DPs obtained by DFT calculation and 95 features that can be obtained from the periodic table. Among them, 425 compounds with direct *E*_g_ validated by PBE-DFT calculations were extracted to construct the *E*_g_ model. The features were chosen by combining the feature importance of the GBR model with the last-place elimination method, and the R^2^, MSE, and MAE tended to be stable and reached the relative optimal value at 32 features. Analysis of the top 10 features revealed that B/B′ and X sites play a key role in *E*_g_ formation. The *PCC*s of the 32 retained features were then calculated, and the features with lower feature importance were deleted when the correlation coefficients between any two features were greater than 0.8. Eventually, 14 features were retained. As a side note, the last-place elimination method (Figure 5b) is found to be RFE in essence, and it seems that researchers tend to use it in conjunction with the GBR model.

### 5.3. Feature Selection for Double Perovskites

The feature selection methods used in the study of DPs also include a single method and a combination of different methods. Wang et al. [40] collected 1747 known DP structures with calculated *E*_g_ values obtained from the MP database to predict the *E*_g_ for rapid screening out suitable DPs. Additionally, based on *E*_g_ values, the target variable was classified into three categories: *E*_g_ less than 1.0 eV, between 1.0 and 2.0 eV, and greater than 2.0 eV. A total of 14 descriptors, including isolated elemental properties and differences between properties, were used as initial features to build the GBDT classification model, and the last-place elimination method was used for feature selection. The top (N − 1) features were selected to perform the next training at the end of each modeling. After visualization of the relationship between the ACC of the model and the number of features, it was found that the ACC of the model reached its optimal value when nine features were selected, with an ACC of ~92%. The important analysis of the features leads to the inference that the design of the B- and B′-site cation combinations has a significant impact on the value of *E*_g_ for DPs. Liang et al. [39] developed ML models based on the energy above the convex hull (*E*_hull_) to screen thermodynamically stable lead-free halide DPs. The dataset was assembled from 469 A_2_B′BX_6_-type halide DPs with known labels and *E*_hull_ values, containing 112 stable compounds with *E*_hull_ ≤ 0 and 357 unstable compounds with *E*_hull_ > 0.24 elemental features combined with six algorithms were used to build classification models for stable/unstable perovskites as well as the regression model of *E*_hull_. Based on the SHAP method for feature selection, the XGBoost classification model was optimal when the top 13 features of the SHAP importance ranking were selected for modeling, with an AUC of 0.9551 under a 10-fold CV. For the regression model, the R^2^ of the XGBoost regression model constructed based on the top 13 features was 0.83, which was only 0.01 lower than when all features were used for modeling. After analysis of the importance of the retained features, it can be inferred from the SHAP summary plot that perovskites with lower Shannon’s ionic radii of X and B′-site atoms as well as higher Shannon’s ionic radii of A and B-site atoms tend to have higher stability. The conclusions of the classification and regression models are consistent. 

Gao et al. [100] proposed a search strategy combining ML and DFT calculations to screen lead-free inorganic DPs with suitable *E*_g_ and high stability. The dataset consists of 481 A_2_B(I)B(III)X_6_ DPs and 264 A_2_B(II)B(II)X_6_ DPs with a target property of *E*_g_ and 28 chemical properties associated with the *E*_g_ as initial features. The *PCC* method and the feature importance from the XGBoost algorithm were used together to select features. If the absolute value of *PCC* for a feature pair is greater than 0.8, a feature with lower feature importance will be deleted. A total of 13 features were finally retained for constructing the models, among which the XGBoost model had the best R^2^ of 0.956. The number of valence electrons at the B-site ranks first, and the B′-site polarizability and the B′-X bond energy are relatively important features. The importance ranking of the top 3 features is reliable, which has been confirmed by published papers or could be reasonably explained based on existing theories. Yang et al. [14] discovered potential oxide DPs with narrow *E*_g_ based on the ML method. Firstly, 79 A_2_B′B″O_6_-type oxide DPs and 75 non-perovskites with *E*_g_ values were collected from the experimental literature. A total of 64 atomic parameters and two process conditions were applied as initial features to the classification model of DPs and the *E*_g_ regression model. To perform feature selection, first the mRMR and *PCC* methods were used to rank the initial features and measure the correlation between features, respectively. The lower-ranking features were eliminated if the *PCC* of a feature pair scored higher than 0.95, and finally 49 and 46 features were retained for building classification and regression models, respectively. Further feature selection was then performed for the retained features in combination with classification and regression algorithms to visualize the relationship between the number of features and the evaluation metrics of models including ACC and R. It was found that the highest prediction ACC under LOOCV for the SVC model was 0.959 when the top six features were selected, and when the top 11 features were selected, R under LOOCV for the SVR model reached a peak of 0.916. Further calculations of the *PCC* between the 11 features and *E*_g_ revealed the same conclusion, in agreement with the results of existing studies, that the *E*_g_ of the oxide DPs is mainly influenced by the ions at the B′ and B″ sites.

**Figure 6:**
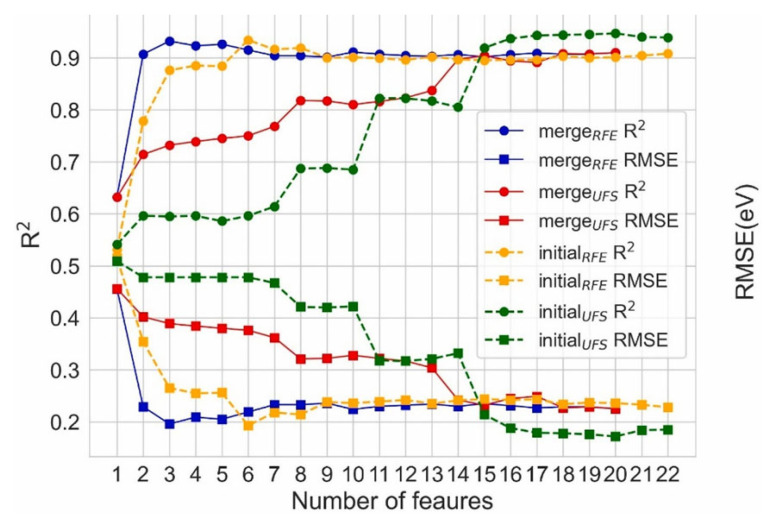
Computed test set RMSE and R^2^ with n-dimension features, with n ranging from 1 to 22 for initial features but from 1 to 20 for merge features. Reprinted with permission from ref. [101]. Copyright 2022 Elsevier.

An interesting case is combining different initial feature sets with different feature selection methods. Liu et al. [101] collected 236 perovskite oxides containing experimental *E*_g_ values from peer-reviewed publications to predict and screen out double perovskite oxides with suitable *E*_g_. There were two feature sets, including the set of initial features, which consists of 42 component features, and the set of merge features, which consists of 20 new features produced from the weighted average of A- or B-site doped element features. The classical nonlinear regression algorithm RF was chosen considering that the *PCC*s between each feature and the *E*_g_ less than 0.5. The univariate feature selection (UFS) and RFE method based on the RF model (RF-RFE) were used for feature selection. Additionally, the feature set and feature selection methods were combined in two ways, i.e., for both the initial feature set and the merge feature set, different numbers of features were selected for modeling using the UFS and RF-RFE methods, respectively, and the optimal models obtained from different combinations were noted as M1, M2, M3, and M4, respectively. When using the RFE method, the prediction performance of the model improves rapidly to the optimal level for both feature sets in the ranges of 1–6 and 1–3, respectively, with an R^2^ of 0.932 and a RMSE of 0.196 eV for a merge feature number of three (Figure 6). Unlike the RFE, when using the UTS method, the prediction performance of the model improves slowly as the number of features increases, with the RMSE for both feature sets achieving the minimum value when the feature dimension was 20. It was found that the A-site ions contribute particularly significantly to the model based on the importance scores of the features in the *M*_1_, *M*_2_, *M*_3_, and *M*_4_ models, and the effect of the A-site ions on the *E*_g_ has been confirmed in studies. According to the *PCC*s between features, it was also found that a feature with a small importance score may not mean including less information because the other selected features contain similar information. 

A point worth pondering is that in the above cases, the feature selection methods chosen for predicting the *E*_g_ of DPs were different, which may be due to the difference in sample size and feature dimensions that led to the different choices finally made after trying different methods.

Here are a few cases of PSCs. Liu et al. [102] used a ML approach to intelligently screen passivation materials that help improve the PCE of PSCs. The dataset had a total of 105 samples, each of which included the interface materials used for the perovskite/hole transport layer (HTL) and the corresponding values of PCE. Feature sets are three types of features extracted from interface materials, perovskites, and the performance of standard devices, including electrotopological-state indexes and cheminformatics, ion ratios in precursor solutions, ion types, and control device performance (C_PCE). The prediction performance of RF models constructed based on different combinations of features showed that the above three types of features played a key role in model performance. Considering that the feature dimension exceeds 300, the 15 most critical features were selected using SHAP and *PCC* methods. The PCE of the modified device and the C_PCE have a high positive correlation with a *PCC* value of 0.84. Additionally, based on the correlation matrix, it can be inferred that excess Pb^2+^ ions in the precursor solution could lead to the high PCE. Four ML algorithms, including linear regression, RF, XGBoost, and neural networks (NN), were used for modeling to map the relationships between the PCE and the 15 selected features. The RF model with the best performance was used for feature importance analysis, and the results showed that C_PCE was the most essential feature for determining PCE, in agreement with the analysis of *PCC*. She et al. [103] used a two-step ML method to predict high-efficiency PSCs with doped electron transport layers (ETLs). The 2006 samples of PSCs were collected from the published literature, and two datasets, which include 1820 and 186 samples, respectively, were constructed for the two-step ML. Additionally, the first dataset was the PCE data of PSCs with undoped ETL, while the second dataset was the efficiency improvement rate (EIR) of PSCs with doped ETL, of which 90 PSCs are doped-SnO_2_-based and 96 are doped-TiO_2_-based. Initial features include the doping element and concentration, the physicochemical properties of dopant elements, and the optoelectronic properties of ETL after doping. The feature engineering of the second dataset was performed based on PSCs of doped-SnO_2_-based and doped-TiO_2_-based, respectively. The RF regression model was first built using all features, and the 16 features were ordered by feature importance. Then, the *PCC* of any feature pairs was calculated, and if the absolute value of the *PCC* was higher than 0.8, the one feature with lower importance in the feature pair was deleted, and the features of doped-SnO_2_-based and doped-TiO_2_-based were finally reduced to 10 and 11. Among the top five features, the Fermi level, CBM, *E*_g_, and conductivity are common features to both SnO_2_ and TiO_2_, as well as the generally accepted factors for ETLs to achieve high PCE of PSCs. Since the *PCC*s between any two retained features are mostly below 0.5, it can be inferred that the redundant features have been successfully removed. Modeling based on the retained features, the RMSE values for SnO_2_ in the training and test sets are 0.05 and 0.04, respectively, while the values of R^2^ are 0.90 and 0.92, which are better than the performance of TiO_2_. 

In addition, it should be noted that all the perovskites in the above literature review section are 3D perovskites. The low-dimensional perovskite materials include 0D, 1D, and 2D perovskites, which are classified depending on the spatial arrangement of octahedra in the form of 0D dots, 1D chains, and 2D layers, respectively [104]. Low-dimensional perovskites have also been widely used in solar cells, light-emitting diodes, and photodetectors due to their flexible structures, excellent photovoltaic properties, and higher stability [105,106]. Among them, 2D perovskites have attracted a lot of attention due to the wide tunability of their photovoltaic properties and excellent stability [107,108]. The (100)-oriented 2D perovskites are the most common, especially the Ruddlesden–Popper (RP) and Dion–Jacobson (DJ) phases [109]. Therefore, a few cases of applications of feature selection in 2D perovskite materials are also briefly described below.

Lyu et al. [110] reported an ML-assisted method to investigate how the dimensionality of lead iodide perovskites was impacted by the structure of organic cations. The dataset is derived from 86 amines reported in the literature for low-dimensional lead iodide perovskites, which were classified according to the dimensionality of the perovskites as “2D” and “non-2D”. A total of 40 initial features were generated by descriptor functions, and 21 features were finally retained after using 0.95 as the threshold for *PCC*s to remove highly correlated features. LR, SVM, KNN, and DT were used to build the classification models, and the LR model with a prediction ACC of 0.82 ± 0.08 on the test set was used in the follow-up study. Feature selection was performed based on the feature coefficients with the $L_{1}$ penalty in the LR model, and four features were finally selected to construct the prediction model. Additionally, it was found that the topological and geometric properties of ammonium cations played a key role in determining the dimensionality. The primary amine with a smaller steric effect index (STEI) is more likely to form 2D perovskites. Due to the eccentricity (Ec) having a feature coefficient of 1.922, it is possible to determine that octylammonium is predicted to form 2D perovskite more readily than cyclooctylammonium. According to the largest ring size (LRS) with a negative feature coefficient, molecules with a bigger ring are likely to produce lower-dimensional perovskite. Hu et al. [111] obtained the adsorption energy of 640 ion/perovskites by first-principles calculations to assess the interaction between 2D A_2_BX_4_ halide perovskites and ions in energy storage applications. The *PCC* method was used for feature selection, and only appropriate features were retained when the *PCC*s of the feature pairs were greater than 0.8 or less than −0.8. A total of 13 features were finally selected from 73 original features. After calculating and ranking the *PCC*s of these 13 features with the adsorption energy, it was found that ion density, melting point, and shell layer had higher rankings, which emphasized the major contributions made by the types of ion adsorbates. A total of six ML algorithms—KNN, Kriging, RF, Rpart, SVM, and XGBoost—were used to build the models, and the XGBoost model had the highest R and R^2^ of 0.968 and 0.93, respectively. Meanwhile, to avoid the bias caused by the *PCC* method, 14 feature ranking methods were selected to comprehensively assess the importance of ion density, ion radius, and first ionization of B-site elements. The different ranking methods consistently show the importance of ion density on the adsorption energy, but the *PCC* method is slightly biased in assessing the importance of atomic radii. Zhang et al. [109] applied the ML method to accelerate the synthetic development of (100)-oriented 2D lead halide perovskites (LHPs). The dataset was derived from 264 crystal structures containing PR and DJ phases in the existing literature, and the feature pool consists of nine features, including the number of protonated nitrogen atoms (q), the radius of the halide ion (r(X)), the distortion of the PbX_6_ octahedral bond length (λ), etc. The Spearman correlation coefficient (SCC) was used to perform univariate feature selection, and the linear correlation coefficient between *λ* and r(X) was found to exceed 0.8, up to 0.91. r(X) was removed because *λ* contained more information, and eight features were finally retained. A total of 26 ML classification models were selected, of which the XGBoost model had the best ACC at 84.4%. The importance of features in the XGBoost model showed that q is the dominant feature. Overall, the electronic, topologic, and geometric properties of the organic amine cations have a significant impact on the crystal structures of 2D LHPs. Using the SHAP method for further feature analysis, it was found that low octahedral bond angle distortion, small inorganic layer spacing, and high octahedral bond length distortion have a significant negative contribution on forming the RP/nRP-phase. It is easy to see that the *PCC* method is still the preferred method, but the comparison results with other ranking methods also show that the *PCC* method sometimes has bias while the SCC method is less common.

Generally speaking, feature selection reduces the dimensionality of the features while maintaining or improving the performance of the model in almost all of the scenarios mentioned above, fully demonstrating the importance of feature selection. In terms of the choice of feature selection methods, *PCC* is the most frequently used method for perovskite materials, but the threshold value selected for filtering highly correlated features varies in different usage scenarios. The mixed feature selection methods are also a common screening strategy. When selecting feature selection methods for one’s own research object, one can first try to use the method with a relatively high frequency, but it should be clear that the effectiveness of the feature selection methods is also closely related to the data quality and the selected algorithm, etc.

## 6. Conclusions and Outlook

In conclusion, feature selection is an essential part of the materials ML workflow. This review briefly introduces the common structures of perovskite materials and the generic descriptor types, as well as the common feature selection methods in the filter, wrapper, and embedded methods. Some of the applications of feature selection in the discovery and design process of perovskite materials based on ML methods are reviewed. It is found that *PCC* in the filter method, RFE in the wrapped method, and tree modeling in the embedded method appear more frequently, whether they are used singly or in combination. From this review, we found that an appropriate feature selection method can reduce model complexity and improve model interpretability to a great extent. Although feature selection has been successfully applied in the materials ML workflow, there is still much room for progress. Here, we tend to propose the following directions for the subsequent application of feature selection in the design and discovery of perovskite materials:

(1) The establishment and improvement of the perovskite materials database: Data is the ‘hardware’ for performing ML, and the quantity and quality of data are the keys to model performance. Compared with other fields, data in the materials field is usually characterized by small size and multiple sources. However, a sample size in a large proportion of materials research articles is less than 1000 or even less than 500. For perovskite materials, a dedicated perovskite database platform to collect data of various excellent properties and perovskite device parameters can be established and made available in a form that adheres to FAIR (findable, accessible, interoperable, and reusable) data principles;
(2) Descriptor construction and sharing: To maximize the accuracy of the model and to avoid situations where the ML results contradict the domain expert knowledge, the descriptors can be constructed manually by combining the material domain knowledge. At the same time, for researchers in non-specialized fields, new descriptors can be constructed automatically by means of SISSO and symbolic regression methods. In addition, to break the professional barriers of different fields and further promote the discovery and design of materials, it is also necessary to establish an online access platform of descriptors corresponding to the database, which can make the professional people focus on doing the professional things to provide a greater possibility for the breakthrough of material properties. Taking perovskite thin film as an example [62,63,64,65,66], we encourage researchers to record more detailed process parameters for preparing high-quality thin films in manuscripts and construct a relevant database of process parameters. The key parameters affecting film quality could be selected by employing suitable feature selection methods based on the database. Then an ML model for quantitative analysis of process parameters and film quality can be constructed, offering the possibility of accelerating the optimization of process parameters and guiding the experimental synthesis of high-quality thin films;
(3) Evaluation and development of feature selection methods: In the application of materials ML workflow, researchers have mostly only objectively stated which methods were used for feature selection, and then model construction and selection based on the selected feature subsets were performed. The selection of methods is, in essence, serving the current data. The input of different feature subsets is the result of different selection methods, so the evaluation and comparison of feature selection methods in conjunction with ML algorithms is also quite an important topic. The development of new feature selection methods for material data can also be considered. Based on some practical experience, the ensemble idea can be used to develop ensemble feature selection methods applicable to materials data, which can ensure the stability of feature subsets and thus have stronger generality.

In summary, with the increase of material requirements and demands as well as the rapid development of intelligent methods, ML will continue to be an important tool for other materials. The feature selection, as a key part of the ML workflow, will also receive more attention in the discovery and design process of perovskite materials via ML.

## Figures and Tables

**Table 1:** Commonly used materials databases, including perovskites.

Name	URL	Data Type
The Perovskite Database Project (PDP)	https://www.perovskitedatabase.com (accessed on 19 March 2023)	Exp.
Open Quantum Materials Database (OQMD)	http://www.oqmd.org/ (accessed on 19 March 2023)	Comp.
Materials Project (MP)	https://materialsproject.org/ (accessed on 19 March 2023)	Comp.
Computational Materials Repository (CMR)	https://cmr.fysik.dtu.dk/ (accessed on 19 March 2023)	Comp.
The Inorganic Crystal Structure Database (ICSD)	https://icsd.fiz-karlsruhe.de/index.xhtml (accessed on 19 March 2023)	Exp.
Materials Platform for Data Science (MPDS)	https://mpds.io/#modal/menu (accessed on 19 March 2023)	Comp. and Exp.
Automatic-FLOW for Materials Discovery (AFLOW)	http://www.aflowlib.org/ (accessed on 19 March 2023)	Comp.

## Data Availability

All the data of the examples could be obtained from the corresponding references.
